# Outcomes of Cardiac Resynchronization Therapy with Image-Guided Left Ventricular Lead Placement at the Site of Latest Mechanical Activation: A Systematic Review and Meta-Analysis

**DOI:** 10.1155/2022/6285894

**Published:** 2022-05-20

**Authors:** Nancy M. Allen LaPointe, Fatima Ali-Ahmed, Frederik Dalgaard, Andrzej S. Kosinski, Gillian Sanders Schmidler, Sana M. Al-Khatib

**Affiliations:** ^1^Department of Medicine, Duke University School of Medicine, Durham, NC 27710, USA; ^2^Duke-Margolis Center for Health Policy, Duke University, Durham, NC 27708, USA; ^3^Department of Cardiology, Mayo Clinic, Rochester, MN 55902, USA; ^4^Department of Cardiology, Herlev and Gentofte Hospital, Hellerup, Denmark; ^5^Department of Biostatistics and Bioinformatics, Duke University School of Medicine, Durham, NC 27710, USA; ^6^Duke Clinical Research Institute, Durham, NC 27710, USA; ^7^Department of Population Health Sciences, Duke University School of Medicine, Durham, NC 27710, USA; ^8^Division of Cardiology, Duke University Medical Center, Durham, NC 27710, USA

## Abstract

**Aim:**

To assess evidence for an image-guided approach for cardiac resynchronization therapy (CRT) that targets left ventricular (LV) lead placement at the segment of latest mechanical activation.

**Methods:**

A systematic review of EMBASE and PubMed was performed for randomized controlled trials (RCTs) and prospective observational studies from October 2008 through October 2020 that compared an image-guided CRT approach with a non-image-guided approach for LV lead placement. Meta-analyses were performed to assess the association between the image-guided approach and NYHA class improvement or changes in end-systolic volume (LVESV), end-diastolic volume (LVEDV), and ejection fraction (LVEF).

**Results:**

From 5897 citations, 5 RCTs including 818 patients (426 image-guided and 392 non-image-guided) were identified. The mean age ranged from 66 to 71 years, 76% were male, and 53% had ischemic cardiomyopathy. Speckle tracking echocardiography was the primary image-guided method in all studies. LV lead placement within the segment of the latest mechanical activation (concordant) was achieved in the image-guided arm in 45% of the evaluable patients. There was a statistically significant improvement in the NYHA class at 6 months (odds ratio 1.66; 95% confidence interval (CI) [1.02, 2.69]) with the image-guided approach, but no statistically significant change in LVESV (MD −7.1%; 95% CI [−16.0, 1.8]), LVEDV (MD −5.2%; 95% CI [−15.8, 5.4]), or LVEF (MD 0.68; 95% CI [−4.36, 5.73]) versus the non-image-guided approach.

**Conclusion:**

The image-guided CRT approach was associated with improvement in the NYHA class but not echocardiographic measures, possibly due to the small sample size and a low rate of concordant LV lead placement despite using the image-guided approach. Therefore, our meta-analysis was not able to identify consistent improvement in CRT outcomes with an image-guided approach.

## 1. Introduction

CRT has been shown to improve outcomes in heart failure patients; however, even among the carefully selected patients for whom clinical trials and subsequent guidelines support the use of CRT, not all patients realize the full benefits of this therapy [1–3]. The left ventricular (LV) lead placement has been identified as an important factor for CRT response [4–7]. In addition to avoiding placement in an apical segment, placing the LV lead in the LV segment with the latest mechanical activation that is free from transmural scarring has been suggested [5, 8, 9]. Previous studies using speckle tracking echocardiography (STE) to identify and target the LV segment of latest mechanical activation showed promising results but were relatively small studies, including 1 to 2 centers with unclear generalizability [10, 11]. Thus, the question remains as to whether a personalized approach to LV lead placement that uses cardiac imaging to identify and target the LV site of the latest mechanical activation can improve CRT outcomes. To address this question, we conducted a systematic literature review to identify randomized controlled studies or prospective observational studies of image-guided approaches for LV lead placement and performed meta-analyses to assess the association between the image-guided approach and CRT outcomes.

## 2. Methods

This review was conducted as part of a National Heart, Lung, and Blood Institute-funded project to synthesize evidence related to CRT and to identify and prioritize clinical and policy evidence gaps [12]. To address one component of the evidence gap regarding the extent and/or location of LV mechanical dyssynchrony predicting CRT outcomes, we conducted a systematic literature review for studies evaluating CRT outcomes in patients with an image-guided approach for LV lead placement within the LV segment with the latest mechanical activation versus a non-image-guided approach. The Preferred Reporting Items for Systematic Reviews and Meta-Analyses (PRISMA) statement was followed [13].

### 2.1. Literature Search Strategy

A literature search was conducted using PubMed and Embase for randomized controlled trials (RCT) or prospective studies of CRT. The MeSH and Emtree terms “cardiac resynchronization therapy” and “cardiac resynchronization therapy device” were used in addition to title and abstract searches for “cardiac resynchronization therapy,” “cardiac resynchronization therapy,” “atrio biventricular pacing,” “atrio-biventricular pacing,” “biventricular pacing,” or “biventricular pacemaker.” Results were limited to English language articles and human studies with publication dates between October 2008 and October 2020.

### 2.2. Study Selection, Abstraction, and Bias Assessment

Two reviewers independently screened all identified titles and abstracts. Publications that met the selection criteria as determined by either reviewer were moved on to full-text review. In addition to being either an RCT or prospective observational study in humans and published in English, all studies had to include the following: the use of CRT in eligible inpatient or outpatient heart failure patients; an assessment of the location and/or extent of LV mechanical dyssynchrony by any imaging method; a report of CRT outcomes of interest in relation to the LV mechanical dyssynchrony. CRT outcomes of interest included all-cause mortality, heart failure mortality, heart failure hospitalization, ventricular arrhythmias, change in LV dyssynchrony, change in echocardiographic parameters (LV ejection fraction, end-systolic volume or diameter, end-diastolic volume or diameter), NYHA class improvement, 6-minute walk test (6 MWT), ICD shocks, quality of life, device-related adverse events, and any composite of the outcomes. Two reviewers then independently reviewed the full text of each selected publication to confirm that the study met the inclusion criteria. Finally, one investigator reviewed the full text of the selected studies to identify those that compared CRT outcomes in patients with an image-guided approach for LV lead placement at the site of the latest LV mechanical activation to those without an image-guided approach for LV lead placement.

One investigator abstracted study characteristics, patient characteristics, and results, and a second investigator confirmed study applicability and the accuracy of the abstracted data. Abstracted study characteristics included study design, number of study sites, location of study site (s), funding source, publication year, study arms, number of subjects, method of assessing LV mechanical dyssynchrony, imaging method, follow-up period, and outcomes. Patient characteristics included age, sex, ischemic cardiomyopathy versus non-ischemic cardiomyopathy, LBBB, QRS duration, sinus rhythm, LV ejection fraction, NYHA class, and location of LV lead. In addition, if reported, the number of patients whose LV lead was confirmed to have been placed within the LV segment of latest mechanical activation (concordant LV lead placement) or next to the segment of latest mechanical activation (adjacent LV lead placement) in each study arm was also captured. The abstracting investigator and the over-reading investigator independently assessed risk of bias using the revised Cochrane risk-of-bias tool for randomized trials [14]. Discrepancies were resolved through discussion.

### 2.3. Statistical Analysis

Categorical outcomes (≥1 class improvement in the NYHA class) were reported as frequencies with percentages. Continuous values were reported as means with standard deviations (SDs). For LVEF, the mean (SD) of the absolute change from baseline to follow-up was reported. For LVEDV and LVESV, the mean (SD) of the relative change, expressed as a percentage, from baseline was reported. For one study, the mean relative change and standard deviation in LVEDV and LVESV were estimated using the reported mean and standard deviation at baseline and the absolute change from baseline since the relative change was not reported [10].

Meta-analyses were conducted for all outcomes of interest that were reported in three or more included studies. Meta-analyses were performed using a DerSimonian–Laird random-effects model, and we conservatively used the Knapp–Hartung approach to adjust the standard errors of the estimated model coefficients [15, 16]. Categorical outcomes were pooled and presented as an odds ratio (OR) with 95% confidence intervals (CIs). Continuous outcomes were pooled and presented as the mean difference (MD) with 95% CI. Heterogeneity was assessed with Cochrane's *Q* and *I*^2^ indexes and the corresponding *p* value. *I*^2^ values greater than 75% indicate large heterogeneity.

*p* values ≤ 0.05 were considered statistically significant. Analyses were performed using R (version 4.0.2), including the R package “metafor” (version 2.4-0).

## 3. Results

**Figure 1:**
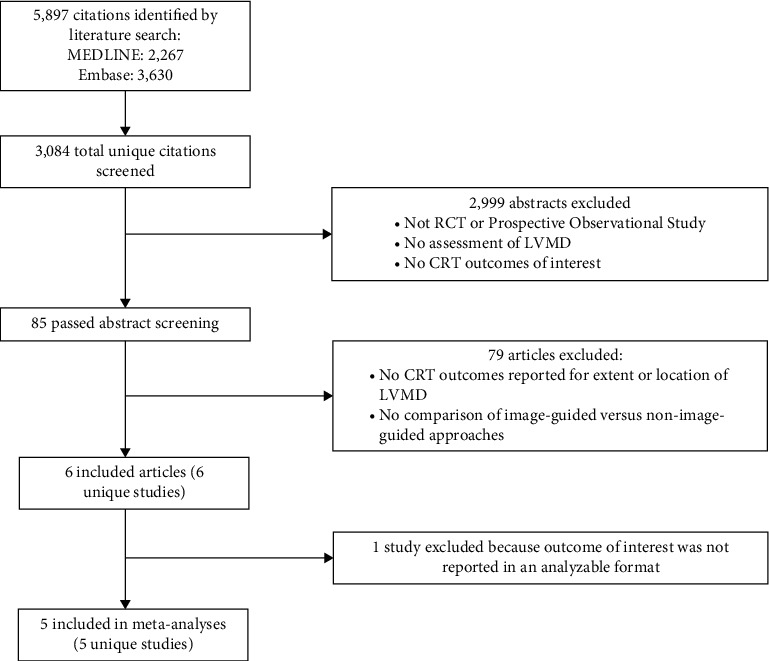
Literature flow diagram. CRT = cardiac resynchronization therapy; LVMD = left ventricular mechanical dyssynchrony; RCT = randomized controlled trial.

From 3084 unique citations identified, a total of 5 studies met the selection criteria for this analysis (Figure 1) [10, 11, 17–19]. All studies were randomized controlled trials, and all but one were single-center studies [10]. While the selected imaging methodology in each study to identify the site of latest mechanical activation was performed on all patients enrolled in the study, the results were provided to the implanting physician only for those patients randomized to the imaging arm. Speckle tracking echocardiogram (STE) was the primary imaging method used in all studies to identify the LV segment of latest mechanical activation and thus the targeted LV segment for LV lead placement. However, three of the studies use multimodal imaging to further refine the targeted segment [17–19]. All three used computed tomography (CT) to visualize coronary sinus branches. In addition, one study used cardiac magnetic resonance (CMR) [17], one used rubidium positron emission tomography [18], and one used single-photon emission computed tomography [19] to identify areas of transmural myocardial scar to avoid LV lead placement. In the control arms, CRT placement was as per the standard of care in three studies [10, 11, 17] and was electrically guided in two studies [18, 19]. However, in one study with the standard-of-care CRT placement, results from the CT and CMR were also made available to physicians [17]. Four of the studies were rated as having a low overall risk of bias [10, 17–19], and one was rated as having some overall risk of bias [11]. Study characteristics are summarized in Table 1.

A total of 818 patients were enrolled in the 5 identified RCTs, with 426 (52%) in the intervention arm and 392 (48%) in the comparator arm. Patient characteristics are presented in Table 2. Characteristics were similar between the control and intervention arms of each study. The mean patient age was greater than 65 years in all studies, and most patients were male (range of 73% to 79%). The proportion of patients with ischemic cardiomyopathy in each study ranged from 46% to 62%, the mean or median QRS duration was over 150 msec in all studies, and all but one study enrolled patients with NYHA classes II, III, and IV. Regardless of the study arm, most LV leads were ultimately placed within a lateral or posterior segment (Table 2). Among 390 evaluable patients in the image-guided arm, 176 (45%) had concordant LV lead placement and 172 (44%) had the LV lead placed in an adjacent segment. In the non-image-guided arm, 117 of 366 (32%) patients had a concordant lead placement and 174 (48%) had an adjacent lead placement.

**Figure 2:**
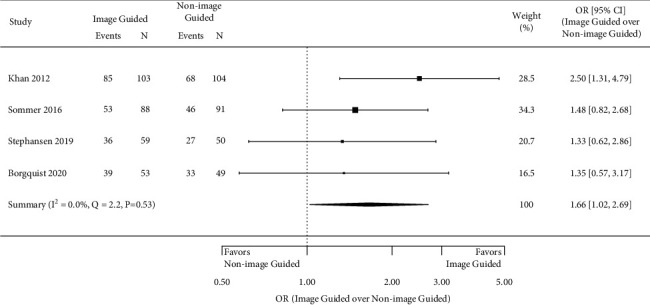
> 1 NYHA class improvement at 6 months.

**Figure 3:**
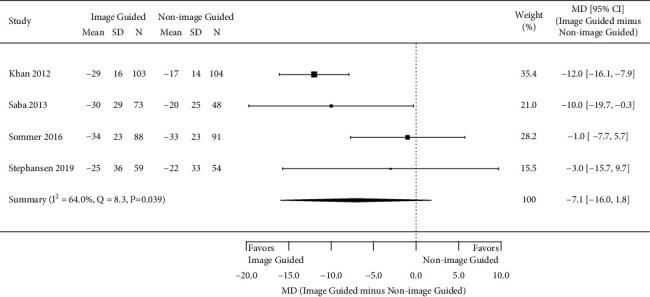
Mean relative reduction in LVESV at 6 months.

**Figure 4:**
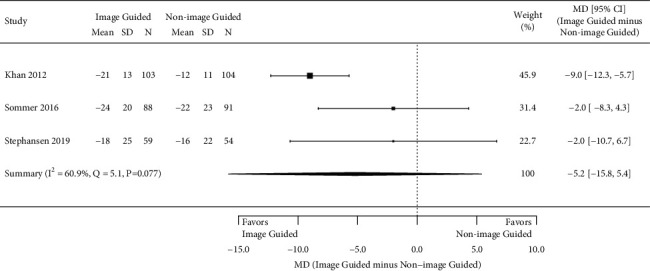
Mean relative reduction in LVEDV at 6 months.

**Figure 5:**
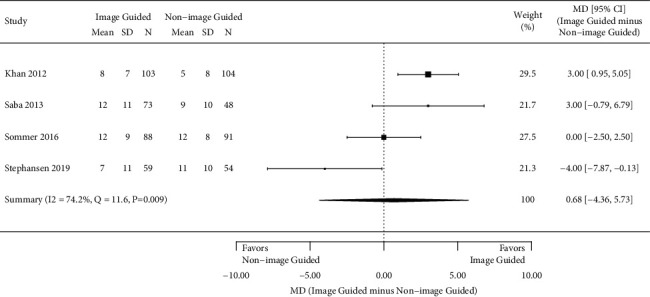
Mean absolute increase in LVEF at 6 months.

All outcomes of interest reported in each study are listed in Table 1; however, only four outcomes of interest were reported in three or more studies and therefore analyzed in this study. In all studies, patients and the physicians assessing outcomes were blinded to the assigned treatment arm. Four studies assessed the number of patients with ≥1 class improvement in the NYHA class, 6 months after CRT placement [10, 17–19]. The meta-analysis showed a statistically significant greater odds of improvement in NYHA class in those with an image-guided approach (pooled OR 1.66, 95% CI 1.02 to 2.69; *I*^2^ = 0.0%, *p*=0.53) (Figure 2). Figures 3–5 present the results from the three other meta-analyses. There were no statistically significant differences between image-guided and non-image-guided approaches in terms of relative reduction in LVESV (mean difference 7.10%, 95% CI −16.00 to 1.80; *I*^2^ = 64.0%, *p*=0.039), relative reduction in LVEDV (mean difference −5.20%, 95% CI −15.80 to 5.40; *I*^2^ = 60.9%, *p*=0.077), or absolute increase in LVEF (mean difference 0.68, 95% CI −4.36 to 5.73; *I*^2^ = 74.2%, *p*=0.009) at 6 months after CRT implant.

## 4. Discussion

In our meta-analysis, we found a statistically significant improvement in the NYHA class with the image-guided approach as compared to the non-image-guided approach; however, no statistically significant differences in any of the echocardiographic measures were found between the approaches. Mortality and heart failure hospitalization outcomes were reported in four of the five included studies; however, because the reported outcome measures were inconsistent across studies and/or the number of events in each study arm could not be ascertained from the publication, meta-analyses for these outcomes were not feasible. The results of our meta-analysis are not consistent with a prior meta-analysis; however, as described in the following, our study provides an expanded and more contemporary perspective [20]. In addition, the five RCTs included in our study had differences in study enrollment criteria and components of the intervention and/or comparator arms, leading to some heterogeneity; however, these trials mirror the evolution of technology and guideline recommendations for CRT. Lastly, the proportion of patients in each study who achieved LV lead placement in the targeted segment varied, as did the rate of patients in the comparator arms who fortuitously had the LV lead placed in the LV segment of latest activation without a scar. This may have resulted in a diminished added value with the imaging-guided approach. Therefore, our meta-analysis was not able to identify consistent improvement in CRT outcomes with an image-guided approach.

The systematic review and meta-analysis conducted by Jin et al. included two studies using speckle tracking echocardiography to identify the target for optimal LV lead placement (Targeted Left Ventricular Lead Placement to Guide CRT (TARGET) and Speckle Tracking Assisted Resynchronization Therapy for Electrode Region (STARTER) studies) and one study using intracardiac echocardiography (ICE) during LV lead implantation to select the best placement option [10, 11, 20, 21]. Jin et al. concluded that the image-guided approach was associated with a statistically significant greater CRT response (OR 2.10; 95% CI 1.43 to 3.07), improvement in LVEF (MD 3.46; 95% CI 1.91 to 5.01), and reduction of LVESV (MD −20.36; 95% CI −27.82 to −12.90) at 6 months [10, 11, 20, 21]. CRT response was defined differently in the three included studies, and therefore, this endpoint was not included in our analysis. In addition, we excluded the study using ICE because it did not meet our inclusion criteria for an image-guided approach that identified a specific target for LV lead placement [21]. However, our analysis included three additional studies that were published after the meta-analysis by Jin et al. [17–19]. These newer studies included multimodal imaging approaches in the intervention arm, and two of them included an electrically guided approach as the comparator. The results from these three studies, in contrast to TARGET and STARTER, largely showed no difference in CRT outcomes between the image-guided and non-imaged arms. Therefore, the results of our study were inconsistent with that conducted by Jin et al. but may provide a more contemporary perspective.

The five RCTs in our study were published over an approximately 8-year time span, and thus, patient selection criteria and components of the image-guided or comparator arms evolved with technology and guidelines during this time. TARGET and STARTER were the first RCTs to address whether an image-guided approach for LV lead placement can improve CRT outcomes [10, 11]. Patients with a QRS duration of ≥120 milliseconds (ms) without considering the presence of LBBB were enrolled. In fact, neither study presents the number of patients with LBBB. However, subsequent studies, following the evolution of clinical practice guidelines, enrolled a high proportion of patients having LBBB, for whom a standard approach targeting the posterior or posterolateral LV segment may be more likely to be the segment of latest mechanical activation [17–19]. In addition, TARGET and STARTER used STE as the sole imaging method. Subsequent studies incorporated multimodal imaging into the intervention arm to better identify transmural scar and coronary anatomy [17–19]. Likewise, the components of the comparator arm evolved over time to include electrically guided approaches and/or some imaging results to inform on coronary anatomy [17, 18]. This evolution in methodology may have resulted in greater heterogeneity among studies and diminishment of the value-added response with an image-guided approach. However, these newer studies more closely represent contemporary capabilities and the importance of this issue, as further reinforced by the recent initiation of a small RCT in the Netherlands comparing CRT outcomes in those with real-time image-guided LV lead placement using CMR feature tracking versus those with the standard-of-care LV lead placement with electrical guidance [22].

Lastly, the primary goal of an image-guided approach is to place the LV lead within the LV segment with latest mechanical activation that is free of scar. While electrical guidance and programming are important components for CRT outcomes, those alone are not likely able to overcome issues associated with a less-than-optimally placed LV lead [22, 23]. Imaging to identify the site of latest mechanical activation was conducted in all patients in all of the included studies; however, the imaging data were only provided to the implanting physician for those patients in each study who were randomized to the image-guided study arm. Therefore, post hoc, the number of patients who ultimately had their LV lead placed at the site of latest mechanical activation (concordant), in the segment adjacent to the segment with the latest mechanical activation (adjacent), and distant from the segment of the latest mechanical activation (discordant) in both study groups was ascertainable and was reported in all of the included studies. The proportion of patients who achieved a concordant LV lead varied among studies with the highest proportion at 61% in TARGET and the lowest proportion at 21% in the study by Borgquist et al. In addition, the proportion of patients with the fortuitous placement of the LV lead within the segment with the latest mechanical activation, free of scar, in the comparator arms also varied, with the highest proportion at 45% in TARGET and the lowest proportion at 12% in STARTER. In a separate meta-analysis, we also found a statistically significant association between CRT outcomes and concordant or adjacent LV lead placement versus discordant LV lead placement [24]. Therefore, the potential lack of differentiation in concordant LV lead placement between study arms may have also diminished the effect from the intervention and merit further study.

There are several limitations to this study, including the relatively small number of studies eligible for inclusion and the small number of total patients. All but one study was a single-center study; however, the included multicenter study only included 2 sites. Therefore, performance of the intervention and the results may not be generalizable. There was also considerable heterogeneity identified in the analyses for LVEF and LVESV. As described above, changes in guideline recommendations and technology over time resulted in potentially significant differences in study populations and/or LV lead implantation techniques, resulting in heterogeneity across studies and/or within studies. Lastly, the study by Borgquest et al. was terminated early due to equivocal results between study arms, potentially introducing bias [17].

In conclusion, our meta-analysis found that an image-guided CRT approach was associated with improvement in the NYHA class but not echocardiographic measures. Therefore, our meta-analysis was not able to identify consistent improvement in CRT outcomes with an image-guided approach. While the small sample size and potential lack of differentiation between study arms in the achievement of concordant LV lead placement may partially explain the equivocal findings, it is also important to note that optimal LV lead placement alone may not fully address the complexity of heart failure management with CRT. Other factors such as device optimization, percentage of biventricular pacing, and arrhythmia burden may also need to be considered and integrated into any future strategic approach for CRT.

## Figures and Tables

**Table 1:** Summary of study characteristics.

First author (year) Study name	Study design, study sites, and locations	Image-guided method	Study arms (*N*)	Outcomes of interest	Risk of bias A B C D E F G
Khan [10] (2012) TARGET	RCT (1 : 1) 2 sites United Kingdom	2D Echo/STE	Image-guided: 110 enrolled; 103 completed SOC: 110 enrolled; 104 completed	Primary: ≥ 15% reduction in LVESV at 6 months Other: ≥1 improvement in the NYHA class, change in the NYHA class, relative change in LVESV, absolute change in LVEF, absolute change in LVEDV—all at 6 months	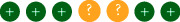
Saba [11] (2013) STARTER	RCT (3 : 2) 1 site United States	2D Echo/STE	Image-guided: 110 enrolled; 73 completed SOC: 77 enrolled; 48 completed	Primary: all-cause mortality or HF hospitalization (mean follow-up 1.8 years (±1.3 years)) Other: all-cause mortality (mean 1.8 years follow-up), HF hospitalization (mean 1.8 years follow-up), relative change in LVESV (at 6 months), and absolute change in LVEF (at 6 months)	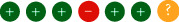
Sommer [19] (2016) Imaging CRT	RCT (1 : 1) 1 site Denmark	2D Echo/STE + SPECT + CT	Image-guided: 89 enrolled; 88 completed Electrically guided: 93 enrolled; 91 completed	Primary: all-cause mortality, HF hospitalization, or improvement (NYHA class and QOL) Other: HF hospitalization or death at 1.8 years, relative change in LVESV, absolute change in LVEF, relative change in LVEDV, and ≥1 improvement in NYHA class—all at 6 months	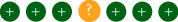
Stephansen [18] (2019) Electro-CRT	RCT (1 : 1) 1 site Denmark	2D Echo/STE + PET + CT	Image-guided: 62 enrolled; 59 completed Electrically guided + CT: 60 enrolled; 54 completed	Primary: absolute change in LVEF at 6 months Other: ≥ 1 improvement in the NYHA class, change in LVESV, change in LVEDV, clinical response (death, HF hospitalization, or improvement (NYHA class and QOL))—all at 6 months	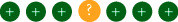
Borgquist [17] (2020)	RCT (1 : 1) 1 site Sweden	2D Echo/STE + CMR + CT	Image-guided: 55 randomized; 53 completed SOC + CT/CMR if available: 52 randomized; 49 completed	Primary: ≥15% reduction in LVESV at 6 months Other: all-cause mortality (within 2 years), all-cause mortality or HF hospitalization (within 2 years), HF hospitalization (within 2 years), ≥1 improvement in NYHA class	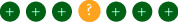

CMR = cardiac magnetic resonance; CT = computed tomography; Echo = echocardiogram; HF = heart failure; LV = left ventricular; LVEDV = left ventricular end-diastolic volume; LVEF = left ventricular ejection fraction; LVESV = left ventricular end-systolic volume; NYHA = New York Heart Association class; Obs = observational; PET = positron emission tomography; QOL = quality of life; SD = standard deviation; SOC = standard of care; STE = speckle tracking echocardiogram; risk of bias: *A* = randomization process, *B* = assignment to intervention, *C* = adhering to intervention, *D* = missing outcome data, *E* = measurement of outcome, *F* = selection of reported results, and *G* = overall (green indicates “low”, yellow indicates “some”, and red indicates “high” risk of bias).

**Table 2:** Summary of patient characteristics.

	Khan et al. [10]	Saba et al. [11]	Sommer et al. [19]	Stephansen et al. [18]	Borgquist et al. [17]
Age					
Image-guided	Median (IQR) 72 (65, 76)	66 (11)	71 (9)	70 (10)	67 (8)
Non-image-guided	72 (64, 80)	67 (13)	71 (9)	72 (8)	70 (8)
All patients	NR	NR	NR	NR	68 (8)
Sex, male					
Image-guided	77%	60%	78%	73%	74%
Non-image-guided	80%	78%	80%	77%	73%
All patients	79%	73%	79%	75%	74%
Ischemic cardiomyopathy					
Image-guided	56%	58%	52%	47%	42%
Non-image-guided	56%	67%	47%	53%	51%
All patients	56%	62%	49%	49%	46%
Sinus rhythm					
Image-guided	100%	75%	NR	NR	89%
Non-image-guided	100%	73%	NR	NR	92%
All patients	100%	74%	NR	NR	90%
QRS duration (ms)					
Image-guided	Median (IQR) 157 (148, 170)	157 (27)	167 (22)	169 (23)	171 (16)
Non-image-guided	159 (146, 170)	162 (27)	165 (22)	170 (17)	169 (22)
All patients	NR	NR	NR	NR	170 (19)
LBBB					
Image-guided	NR	NR	84%^*∗*^	84%^*∗*^	74%
Non-image-guided	NR	NR	88%^*∗*^	93%^*∗*^	74%
All patients	NR	NR	86%^*∗*^	89%^*∗*^	74%
NYHA class					
Image-guided					
II	0	16%	49%	66%	26%
III	86%	64%	49%	31%	68%
IV	14%	20%	1%	3%	6%
Non-image-guided					
II	0	8%	43%	58%	25%
III	85%	71%	52%	40%	57%
IV	15%	21%	5%	2%	18%
All patients					
II	0	13%	46%	62%	26%
III	85%	67%	51%	35%	63%
IV	15%	20%	3%	2%	12%
LVEF (%)					
Image-guided	Median (IQR) 23 (19, 28)	26 (6)	25 (6)	31 (8)	23 (10)
Non-image-guided	24 (18, 29)	26 (7)	24 (6)	29 (8)	23 (12)
All patients	NR	NR	NR	NR	23 (11)
LV lead placement					
Image-guided				NR	
Anterior	3%		5%		22%
Anterolateral	0				
Lateral	46%		47%		45%
Posterolateral	0		0		4%
Posterior	35%		44%		25%
Inferior	12%	NR	5%		4%
Non-image-guided				NR	
Anterior	6%		2%		22%
Anterolateral	0				
Lateral	47%		42%		43%
Posterolateral	0		0		4%
Posterior	38%		56%		28%
Inferior	6%	NR	0		4%
All patients				NR	
Anterior	5%	0%	3%		22%
Anterolateral	0	16%	0		0
Lateral	46%	42%	44%		44%
Posterolateral	0	34%	0		4%
Posterior	37%	8%	50%		26%
Inferior	9%	0%	2%		4%

Values are mean (standard deviation), unless otherwise specific. ^*∗*^100% when including patients with chronic right ventricular pacing and QRS ≥180 ms per entry criteria at baseline. A = anterior; AL = anterolateral; I = inferior; IQR = interquartile range; LBBB = left bundle branch block; LV = left ventricle; LVEF = left ventricular ejection fraction; NYHA = New York Heart Association; NR = not reported.

## Data Availability

The data underlying the findings are included in the article.

## Additional Points

*Summary and Highlights.* Five randomized controlled trials were identified that compared CRT outcomes with an image-guided approach versus a non-image-guided approach; however, the components of the image-guided approach and non-image-guided approach were not identical in all studies. Achievement of a left ventricular (LV) lead placement within the LV segment with the latest mechanical activation (concordant) occurred in 45% of evaluated patients with the image-guided approach but also fortuitously occurred in 32% of evaluated patients with the non-image-guided approach. The image-guided approach for placement of the LV lead for CRT within the LV segment with the latest mechanical activation was associated with a statistically significant greater odds of the NYHA class improvement at 6 months than the use of a non-image-guided approach. No differences in echocardiographic measures at 6 months following the CRT implant were found with the image-guided approach as compared to a non-image-guided approach. Therefore, this meta-analysis was not able to identify consistent improvement in CRT outcomes with an image-guided approach.
